# Collision-spike Sputtering of Au Nanoparticles

**DOI:** 10.1186/s11671-015-1009-x

**Published:** 2015-08-06

**Authors:** Luis Sandoval, Herbert M Urbassek

**Affiliations:** Theoretical Division, Los Alamos National Laboratory, Los Alamos, New Mexico, 87545 USA; Physics Department and Research Center OPTIMAS, University Kaiserslautern, Erwin-Schrödinger-Straße, Kaiserslautern, D-67663 Germany

**Keywords:** Molecular dynamics, Sputtering, Nanoparticles, Clusters

## Abstract

Ion irradiation of nanoparticles leads to enhanced sputter yields if the nanoparticle size is of the order of the ion penetration depth. While this feature is reasonably well understood for collision-cascade sputtering, we explore it in the regime of collision-spike sputtering using molecular-dynamics simulation. For the particular case of 200-keV Xe bombardment of Au particles, we show that collision spikes lead to abundant sputtering with an average yield of 397 ± 121 atoms compared to only 116 ± 48 atoms for a bulk Au target. Only around 31 % of the impact energy remains in the nanoparticles after impact; the remainder is transported away by the transmitted projectile and the ejecta. The sputter yield of supported nanoparticles is estimated to be around 80 % of that of free nanoparticles due to the suppression of forward sputtering.

## Background

Ion irradiation of constrained systems, such as nanoparticles (NPs), nanostructured surfaces, or two-dimensional systems, has recently received increased interest [1]. Ion impact of NPs finds applications in the sputtering of aerosols [2] and in the irradiation of dust particles in space or in a plasma environment [3, 4]. In addition, dedicated experiments have become possible where NPs are supported on a surface and irradiated by an ion beam. Such experiments allow to modify the NPs [5, 6] and also to measure the sputter yield by monitoring the size change of the NP as a function of ion dose. Such experiments have been performed by Klimmer et al. [6] (Ziemann, P. Private communication 2010. Unpublished) and more recently by Greaves et al. [7]. Yang et al. [8] determined the sputter yield of Au NPs in the size range of 10–100 nm under 20-keV C _60_ impact.

From the theoretical point of view, sputtering of spherical NPs has been studied most extensively. Molecular dynamics (MD) computer simulation can shed light on this phenomenon. Many previous simulations focused on Au targets and studied exemplary cases, such as 100-keV Au → Au (sphere radius *R*=4 nm) [9, 10] or 16- and 64-keV Au → Au (*R*=10 nm) [11]. Järvi et al. [12, 13] analyzed the sputtering of Au NPs with radii up to 8 nm by 25-keV Ga ions using MD computer simulation.

Sputtering of NPs by collision cascades can be investigated by analytical theory and—relatively inexpensive—Monte Carlo simulations; in this area, rather encompassing studies have been carried out. Nietiadi et al. carried out an extensive simulation study of the sputter yield as a function of sphere radius *R* [14, 15], using both Monte Carlo and exemplary MD simulations. Their study focused on 20-keV Ar impacts on amorphous Si spheres; here, sputtering proceeds mostly by the ballistic collision-cascade mechanism [16], and the contribution of collision spikes is minor. They showed that sputtering has a maximum as a function of sphere size when the ion penetration depth is of the order of the sphere radius; these results corroborate the earlier findings of Järvi et al. [12, 13]. In a recent publication, Urbassek et al. [17] extended these collision-cascade results to arbitrarily shaped NPs.

Greaves et al. [7] present experiments and accompanying computer simulations for the sputter yield enhancement during the bombardment of Au nanorods under 80-keV Xe irradiation. They show strong enhancements of the sputter yield compared to the sputtering of a bulk Au target and emphasize the role of cluster emission for high-yield events and of channeling for low-yield events.

Here, we analyze the sputtering of spherical Au NPs of radius *R*=12.4 nm by 200-keV ion impact. This system corresponds to the experimental study of Klimmer et al. [6] (Ziemann, P. Private communication 2010. Unpublished) who investigated the burrowing of supported NPs into the substrate under continuous ion irradiation. Since this is a clear case of spike sputtering, MD is used for simulation. We demonstrate the large variety of sputter events occurring. The sputter yield of supported NPs will be smaller than that of free NPs due to the suppression of forward sputtering.

## Methods

Using MD simulation, we study the sputtering of Au NPs by 200-keV Xe impact. The NP radius is set to *R*=12.4 nm and the spherical cluster consists of 463,878 atoms. The Xe ion impacts on a local (111) facet. The local angle of incidence is varied between *θ*=0° (central impact) and 60°. In total, a number of 50 events are simulated. We follow the simulations for 100 ps.

**Fig. 1:**
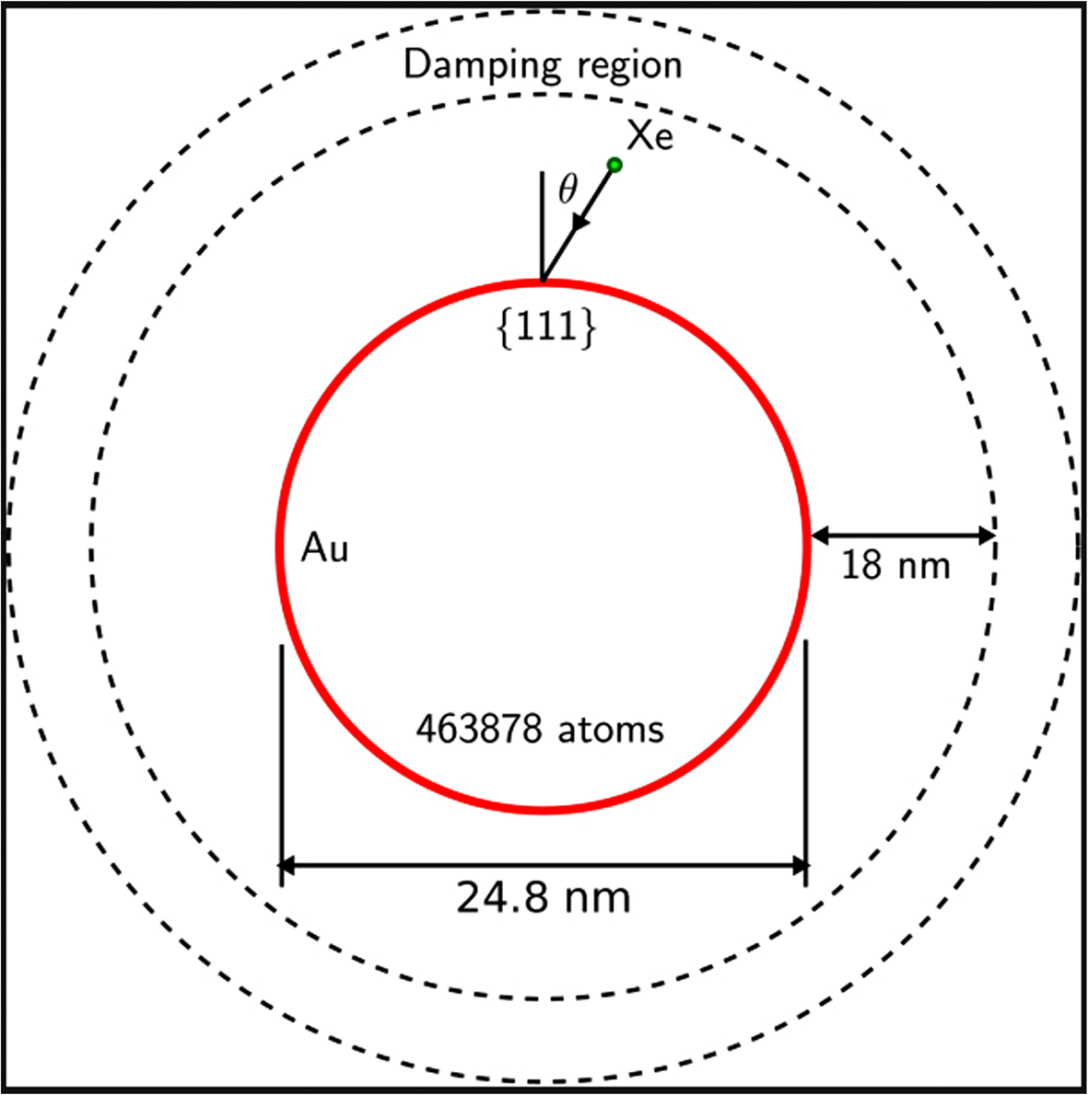
Sketch of the simulation setup. The Au spherical NP consists of 463,878 atoms and has a diameter of 24.8 nm. The 200-keV Xe projectile hits the surface at an angle of *θ* to the local surface normal on a local (111) facet. Sputtered atoms reach a damping zone as soon as they are a distance of 18 nm away from the original cluster surface

In order to increase the efficiency of the parallelized code, we defined an external shell which slowly stops the ejecta by means of a drag term in the dynamics (see Fig. 1). The magnitude of the drag term and the thickness of the corresponding drag region are chosen in such a way that the sputtered clusters are kept apart of each other.

The Au-Au interaction is modeled by a many-body potential which accurately reproduces the melting temperature of Au [18–20]. Note that the choice of the interaction potential is of prime importance for modeling sputtering phenomena under spike conditions [21–23]. Towards high energies, the potential has been splined to the Ziegler-Biersack-Littmark (ZBL) potential [24]. The Xe ion interacts only by the repulsive ZBL potential with the Au atoms.

We model electronic stopping by a velocity-proportional friction force *F*=*nkv*, where *n* is the atomic number density of Au and *v* is the atom velocity. The constant *k* is taken from the Lindhard-Scharff formalism [25, 26], which gives *k*=0.506 eVÅps for the Au-Au and 0.388 eVÅps for the Xe-Au interaction. Usually, electronic stopping is not applied to slow atoms, with a kinetic energy below a cut-off, *E*_*c*_, since up-to-date estimates of electron-phonon coupling—which describes the low-energy transfer of electrons to atoms—give orders of magnitude smaller energy transfer than the Lindhard-Scharff prediction [27]. While the exact value of *E*_*c*_ is unknown—but expected to be in the range of a few eV [28, 29]—we adopt a value of *E*_*c*_=3.9 eV, equal to the cohesive energy; in collision-cascade sputtering, atoms with a smaller kinetic energy are never sputtered. For reference, we also performed a set of simulations for vanishing electronic losses. In order to investigate the role of electronic stopping in more detail, we performed further simulations with smaller *E*_*c*_, including *E*_*c*_=0 (where electron losses apply to all moving atoms in the NP), but only for perpendicular incidence.

We note, however, that all energy put into the electronic system of the NP will thermalize there and thus eventually flow back to the atomic system. This effect will raise the temperature of the NP after sputtering; we do not include this effect in our simulations presented here.

## Results and Discussion

As a first orientation of the geometry of the energy deposition by the projectile in the NP, we use data of the range distribution provided by the SRIM software [30, 31] and corrected by the factors given in [32]. This gives us the result that—in a bulk medium—the center of the deposited energy distribution is at a depth of *a*=19.9 nm below the surface; it has a longitudinal width of *α*=11.9 nm and a transverse width of *β*=11.1 nm. This means that the deposited-energy profile fills out the NP with radius *R*=12.4 nm, and hence, considerably more abundant sputtering is to be expected than from a semi-infinite flat target. In fact, recent calculations [13, 15] of the dependence of the sputter yield on the NP radius predict sputtering to become maximum when *R*≅*a*; this is the case here.

**Fig. 2:**
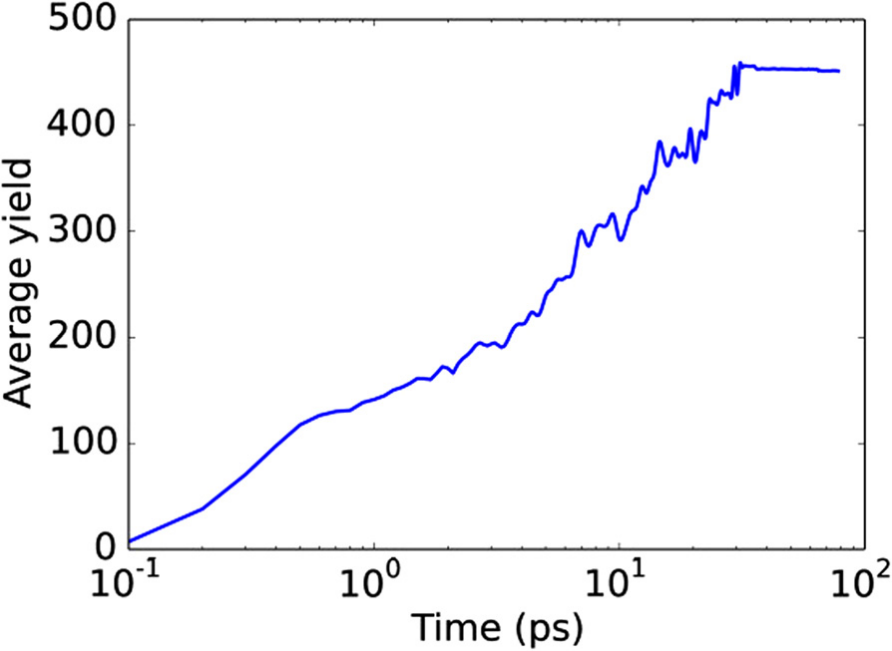
Temporal evolution of the sputter yield. Data obtained as average over central impacts

Figure 2 demonstrates that particle emission proceeds over long time scales, beyond 30 ps. This is typical of spike sputtering: while collision-cascade sputtering is usually terminated after a few ps, sputtering from spikes is accompanied by strong changes in the target surface topography (crater formation), late emission caused by the high temperatures prevailing close to the irradiated surface, and emission of large clusters late after impact [33, 34]. A recent review stresses the importance of both gas-flow emission to the sputtering process and melt flow to crater formation in spike processes [35].

**Fig. 3:**
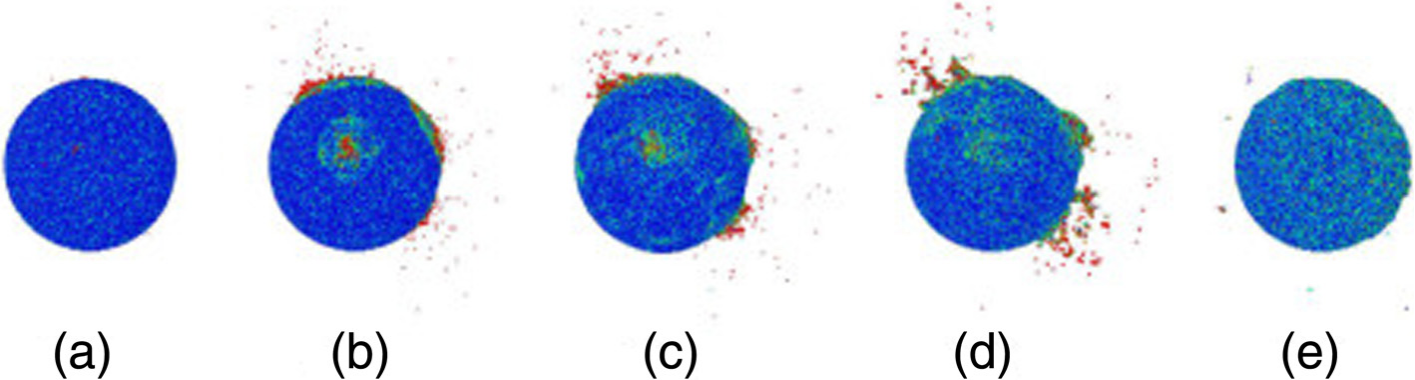
Snapshots of a selected impact event at 0.1 (**a**), 1.0 (**b**), 3.5 (**c**), 10 (**d**), and 100 ps (**e**). Ion impacts at the top at perpendicular incidence. Particles are colored according to their kinetic energy from *blue* (0 eV) to *red* (≥0.4 eV). Final sputtering yield amounts to 1164

**Fig. 4:**
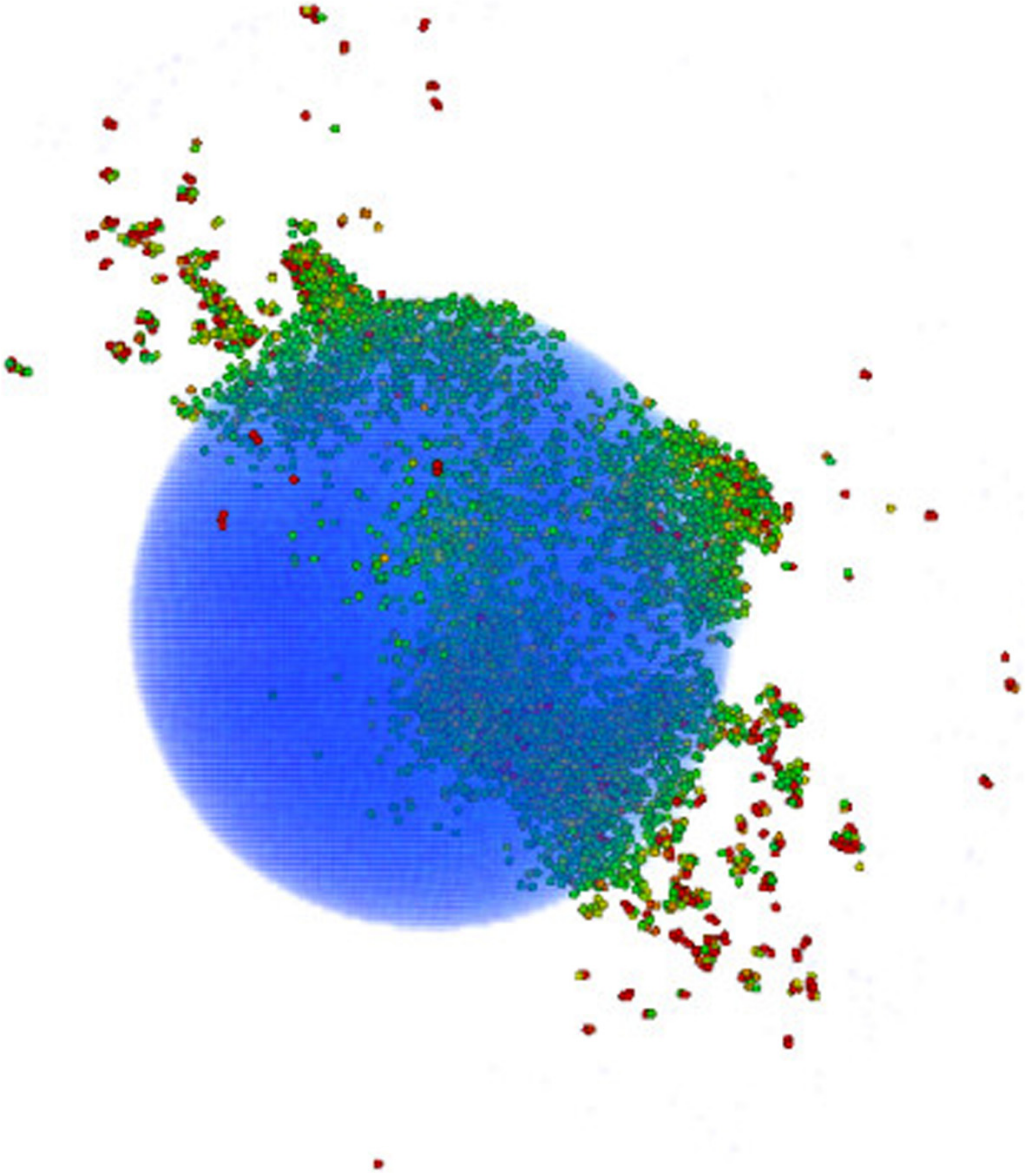
Snapshot of the event shown in Fig. 3 at 11 ps after particle impact. At this time also sideways and forward sputtering have set in. In this presentation, thanks to transparency (*blue particles*), the recoil particles inside the sphere are also seen. Only the particles with a kinetic energy above 0.4 eV are displayed. Particles with kinetic energy between 0.4 and 1 eV are colored from *green* to *light red*, particles above 1 eV in *dark red*

Figure 3 shows the time evolution of a particular impact; in this event, the ion impact was central to the top of the NP with perpendicular impact direction. The abundant ejection starts early, at 1 ps; emission occurs both close to the impact point and at several other places at the surface of the sphere, where the projectile or fast recoils deposited their energy. Emission increases from these spots until 10 ps. At 100 ps, Fig. 3e, sputtering has ceased. Ejection occurs from isolated spots at the NP surface, both in forward and backward direction. Figure 4 shows how sputter emission is connected to energetic recoils within the NP; in this presentation, the sphere has been rendered transparent. We see how several branches of the collision cascade reach to surface spots, and particle emission has started there.

**Fig. 5:**
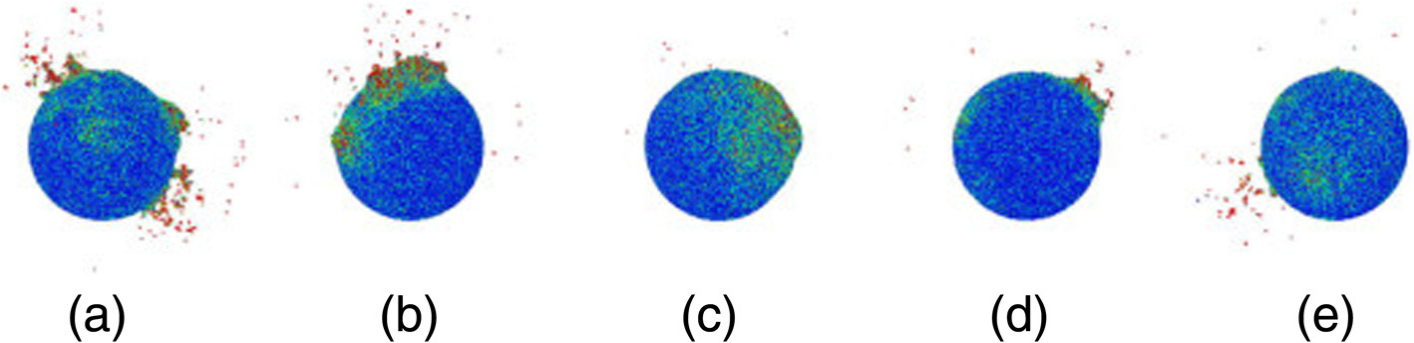
Snapshots of several events (**a**-**e**) at 10 ps after particle impact. Ion impacts at the top at perpendicular incidence angle. Particles are colored according to their kinetic energy from *blue* (0 eV) to *red* (≥0.4 eV). Event (**e**) corresponds to that shown in Fig. 3

We give an impression of the variety of impact events and the sputter emission sites in Fig. 5 which assembles snapshots from different projectile impact points showing the extension of the energy deposition in the NP. The event of Fig. 3 corresponds to Fig. 5a. Fluctuations in energy deposition are strong and so are the ensuing emission profiles. While sometimes only little energy is deposited close to the impact point, Fig. 5c, in other events, energy is deposited both close to the ion impact and at the ion exit point on the NP, Fig. 5a. In most events, a spike is clearly observed in the NP, which is discernible by the high density of particles moving with high energy. We note that kinetic energies above *E*=0.4 eV correspond to a local temperature of above *T*=1550 K, assuming *E*=3*kT*. The zones delineated in Fig. 5 thus correspond to the molten zones in the NP.

This figure shows that the NP is not filled homogeneously with energy by the projectile. This may appear astonishing since the dimensions of the deposited-energy ellipsoid are all comparable to the NP radius. However, it has been known for a long time that individual energy deposition profiles are actually smaller than the average deposited-energy ellipsoid [36], and our snapshots underline this fact for the present case.

**Fig. 6:**
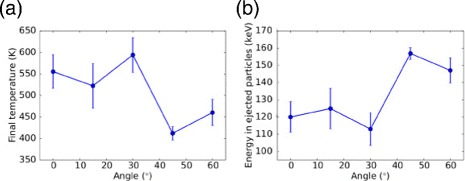
Temperature and energy taken away from NP by emission. **a** Temperature in the irradiated NP at the end of the simulation and **b** energy taken away by the transmitted projectile and sputtered particles as a function of impact angle

We determine the total amount of energy actually deposited in the NP. Figure 6a shows the final temperature in the NP as a function of the impact angle. We see that the temperature has considerably increased above the initial temperature of 300 K. The average temperature increase amounts to 189 ± 32 K; this corresponds to a retained energy in the NP of 22 keV. The energy taken away from the NP by the transmitted projectile and the sputtered particles amount to roughly 138 ± 7 keV, as Fig. 6b demonstrates. However, 40 keV has been deposited in the electronic system of the irradiated NP; it will flow back with a time scale of 98 ps [27] to the atomic system of the NP. Thus, in total, 62 keV is retained in the NP, corresponding to 31 % of the impact energy.

We note that when electronic stopping is ignored, the temperature increase in the atomic system of the NP is higher (349 ± 49 K), since the spikes are longer lived, corresponding to 42 keV, while 158 keV leaves the NP by the transmitted projectile and the sputtered particles. Thus, electronic energy loss helps in localizing impact energy in the NP, since it is eventually refueled to the atomic system. This is a quite different situation from the bombardment of bulk targets, where the energy lost to the electronic system diffuses away.

**Fig. 7:**
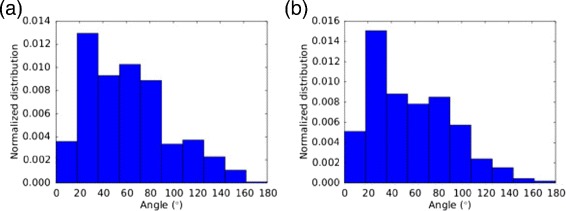
Angular distribution of sputtered particles. **a** Data obtained as average over central impacts. **b** Average over all impacts

Figure 7a presents the angular distribution of sputtered particles for central impacts and Fig. 7b averages over all impacts. The angular distribution, *f*(*𝜗*), is defined such that *f*(*𝜗*) sin*𝜗d𝜗* equals the number of atoms emitted in the angular range (*𝜗*,*𝜗*+*d𝜗*). Thus, isotropic emission from the NP, *f*(*𝜗*)=const, corresponds to a sine distribution in Fig. 7. Both the average over central impacts, Fig. 7a, and the average over all impacts, Fig. 7b, show a more backward-oriented emission. The forward-sputter fraction is 19 % when averaged over all events.

We calculate the average sputter yield, 〈*Y*〉, by averaging over all impact parameters *b* with which ions impact on the NP,

(1) $$  \langle Y \rangle = \frac 1{\pi R^{2}} {\int_{0}^{R}} Y_{R}(b) 2\pi b \, \mathrm{d} b \,.  $$

Here, the impact parameter *b* is determined from the local incidence angle (with respect to the surface normal) *𝜗* by

(2) $$  b = R \sin \vartheta \,.  $$

We obtain an average sputter yield of 〈*Y*〉=397 ± 121 atoms. We also determined the sputter yield of a flat (infinitely thick) Au target under 200-keV Xe impact at normal incidence; it amounts to 116 ± 48 atoms.

This result can be compared with the experimental sputter yield of a flat surface. Szymczak and Wittmaack [37] measured *Y*=50 for (perpendicular) 200-keV Xe impact on a (111) Au surface. For comparison, we also note that the software TRIM (Transport of Ions in Matter) [38] gives a sputter yield of only 27 (Möller, W. Private communication 2014. Unpublished) for the flat surface; since TRIM takes only (ballistic) collision-cascade sputtering into account, this comparison demonstrates that spike sputtering is important already for the sputter emission from a bulk Au target.

The strong (fourfold) increase in sputter emission from the flat target to the NP target is similar to the data reported by Greaves et al. [7] for 80-keV Xe sputtering of Au nanorods. There, MD gave an average sputter yield of 1005. Only four individual experimental results are reported, ranging from 147 to 843.

Monte Carlo simulations show an increase of the sputter yield for nanospheres as compared to that of the bulk target [15]. For the conditions of our system, *a*/*R*=0.623, these predict a yield enhancement of 6.98 for central impacts and of 2.14 as an average over all impacts. While these Monte Carlo simulations have been performed for another system (20-keV Ar on Si), they may be taken as a guideline to estimate the size of the ballistic enhancement effect to be expected. Our simulations show that the enhancement effect for collision spikes—here by a factor of around 4—may exceed that of collision-cascade sputtering.

We performed simulations for perpendicular incidence on the NP and on the flat target for various values of the cut-off energy, *E*_*c*_, for electronic stopping (see Table 1). We see that electronic stopping strongly influences the sputter yields, as was found previously [27]. With increasing cut-off, electronic stopping influences the recoil atoms less, the spikes are less quenched, and sputtering proceeds for a longer time; hence, the sputter yields increase. At cut-off energies *E*_*c*_≤1.5 eV, the values saturate at low levels but increase strongly when *E*_*c*_ reaches the cohesive energy, 3.9 eV. Collision-cascade sputtering would predict that the sputter yield is constant as long as *E*_*c*_≤3.9 eV, the cohesive energy of Au, since only atoms with energies above this threshold are able to overcome the surface barrier and be sputtered. The fact that sputtering depends on *E*_*c*_ below the cohesive energy hence gives evidence of the importance of spike sputtering in this system.

**Table 1:** Dependence of the sputter yield of a flat surface and a NP on the cut-off energy *E*_*c*_ for electronic stopping. Data obtained for perpendicular incidence only

*E*_*c*_	0 eV	1.5 eV	3.9 eV	No electronic stopping
Flat surface	53±7	56±18	116±48	644±309
NP	96±18	101±30	397±121	5096±1804

In experiment, NP sputtering is measured after depositing NPs on a substrate. In such a situation, the sputter yield will depend on the material of the substrate and the exact shape of the NP, which may vary depending on the deposition technique and the interface energy between NP and substrate from spherical over hemispherical to a flat layer. We estimate the sputter yield of a supported NP by assuming that only backsputtering contributes in this situation. Correcting the simulated sputter yield by excluding all forward-sputter events, we arrive at a sputter yield of 81 % of the total sputter yield, *Y*∼322 ± 98.

The experimental sputter yield of Au NPs under 200-keV Xe impact supported on a sapphire surface amounts to 149 ± 47 (Ziemann, P. Private communication 2010. Unpublished). The spherical particles were prepared by a self-assembly process leading to a narrow size distribution. The sputter yields were determined by monitoring the change of the particle size distribution on the surface as a function of ion fluence. The experimental sputter yield is slightly smaller than our MD estimate; including the theoretical and experimental error bars, the predictions are compatible with each other. Several reasons may contribute to this difference. (i) In the range of high temperatures and pressures and low particle densities relevant for the spike region, the interatomic potential is only poorly known; small changes in the interaction potential may affect the sputter yield in the spike regime [18, 28]. (ii) The role of the electronic system under spike conditions is complex. A recent model study [27] of spike sputtering of bulk Au targets showed that the energy loss to the electronic system will withdraw the energy from the atomic system; this may happen so rapidly that the collision spike may be quickly quenched and the sputter yield is reduced to that of the collision cascade. (iii) The interatomic interaction potential in Au depends on the electron temperature. A recent study [39] calculates this dependence and shows that in particular the melting temperature will be increased for high electron temperatures due to the increased electron pressure. It would be worthwhile to continue the present studies by including these coupled effects of the electron and atom system to the simulation of spike sputtering in NPs. (iv) The sputtering of a supported NP is a complex situation; on the one hand, energy can dissipate from the NP to the substrate decreasing the NP sputter yield; on the other hand, also the substrate may become sputtered and lead to a secondary sputtering of the NP. Such situations have up to now been rarely investigated [40]. (v) Finally, artifacts in the experiment may affect the yield measurement. For example, it was recently reported [41] that even a single graphene layer on a metal surface decreases the sputter yield of metal atoms dramatically. If the NPs in the experiments were covered by a graphene (carbon) layer formed by the dissociation of ubiquitous hydrocarbons, this might have reduced the measured yield.

Ion irradiation of nanoparticles leads to enhanced sputter yields if the nanoparticle size is of the order of the ion penetration depth. While this feature is reasonably well understood for collision-cascade sputtering, we explore it in the regime of collision-spike sputtering using molecular-dynamics simulation. For the particular case of 200-keV Xe bombardment of Au particles, we show that collision spikes lead to abundant sputtering with an average yield of 397 ± 121 atoms compared to only 116 ± 48 atoms for a bulk Au target. Only around 31 % of the impact energy remains in the nanoparticles after impact; the remainder is transported away by the transmitted projectile and the ejecta. The sputter yield of supported nanoparticles will be only around 80 % of that of free nanoparticles due to the suppression of forward sputtering.

## Conclusions

Simulation of NP sputtering is conceptually simpler than that of extended targets. Since electrons cannot escape the NP, the energy put by electronic stopping into the electronic system is not lost but remains in the target. Our simulations treat the system of 200-keV Xe impact on spherical Au NPs of *R*=12.4-nm radius as an exemplary case. In this case, the mean depth of energy deposition is situated almost in the center of the NP. Still, only around 20 % of the projectile energy is finally deposited in the NP; the remainder is transported away by the projectile and by the ejecta.

We find an average sputter yield of 397 ± 121, while the sputter yield of a bulk target is only 116 ± 48. Such large yields are due to spike effects which are virtually unquenched since electrons do not carry away the energy.

Around 20 % of the sputtered species is emitted in the forward direction. For supported NPs, this emission will be suppressed since the substrate will prevent ejection. The corresponding correction lowers the sputter yield to values of around 320.
